# Large papillary fibroelastoma of right atrium, an unusual case of respiratory distress in a young man

**DOI:** 10.1186/s13019-021-01526-7

**Published:** 2021-05-29

**Authors:** Yan Le Ho, Pui Fong Ng, Sotheenathan Krishinan, Basheer Ahamed Abdul Kareem

**Affiliations:** 1 Department of Cardiothoracic Surgery, Penang General Hospital, Georgetown, Malaysia; 2 Department of Pathology, Penang General Hospital, Georgetown, Malaysia

**Keywords:** Papillary fibroelastoma, Cardiac tumour, Pulmonary embolism

## Abstract

**Background:**

Papillary fibroelastomas are rare but benign cardiac tumour that are often found on cardiac valvular surfaces. Their clinical manifestations ranging from clinically asymptomatic to substantial complications that are usually secondary to systemic embolism. Multiple theories have been proposed to explain the pathophysiology of its formation.

**Case presentation:**

We reported a rare case of large papillary fibroelastoma in the right atrium of a young gentleman which was complicated with pulmonary embolism. Transthoracic echocardiography identified a large pedunculated mass measuring 3.4cmX3.4cmX2cm in right atrium with stalk attached to interatrial septum. The intracardiac mass was resected surgically, which revealed papillary fibroelastoma in histology examination.

**Conclusion:**

Differential diagnosis of intracardiac masses requires clinical information, laboratory tests and imaging modalities including echocardiography. Incidentally discovered papillary fibroelastomas are treated on the basis of their sizes, site, mobility and potential embolic complications. Due to the embolic risk inherent to intraacardiac masses, surgical resection represents an effective curative protocol in treating both symptomatic and asymptomatic right sided and left sided papillary fibroelastomas, with excellent long term postoperative prognosis.

## Introduction

Cardiac neoplasms are rare compared to other cardiac diseases and tumours of other organs. Most cardiac tumours are benign, and papillary fibroelastoma is the second most common benign cardiac tumour after myxoma, which only accounts for < 10% of all cardiac tumours [1]. This tumour predominantly located on valvular structures. The clinical presentation of papillary fibroelastoma varies from asymptomatic to severe embolic complications.

## Case report

**Fig. 1:**
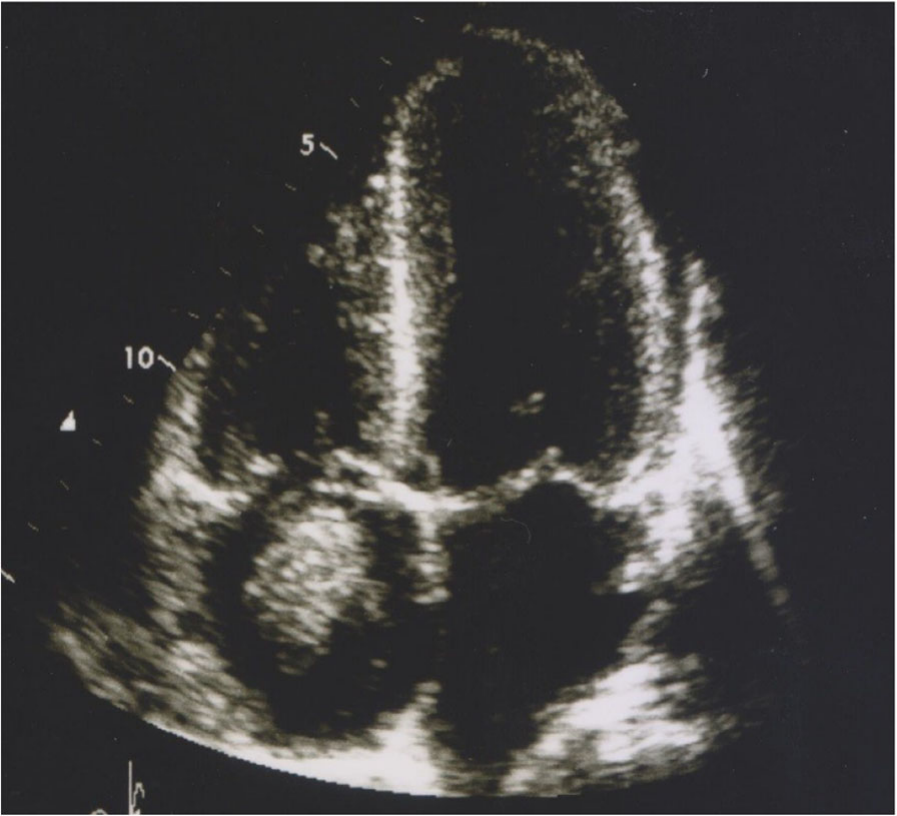
Echogenicity mass (arrow) in right atrium attached by a stalk to interatrial septum

**Fig. 2:**
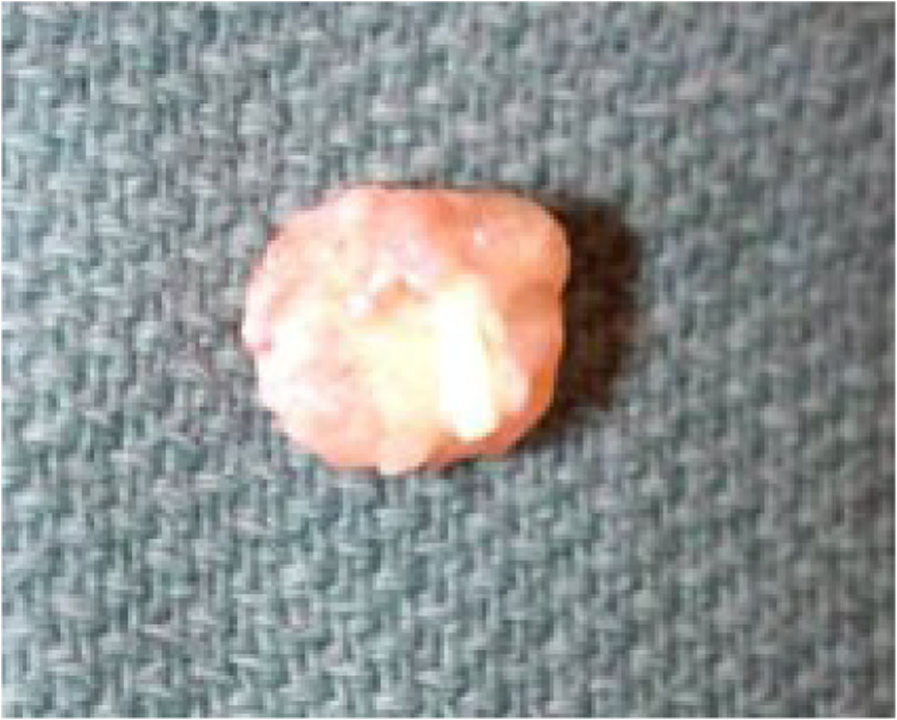
Part of the resected specimen revealed a soft gelatinous mass with flower-like appearance

**Fig. 3:**
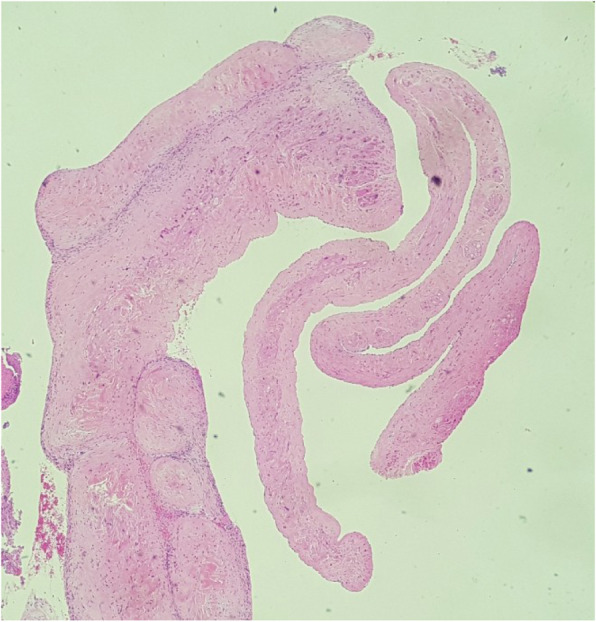
Section shows some viable delicate branching papillary fronds, H&E, X40 magnification

**Fig. 4:**
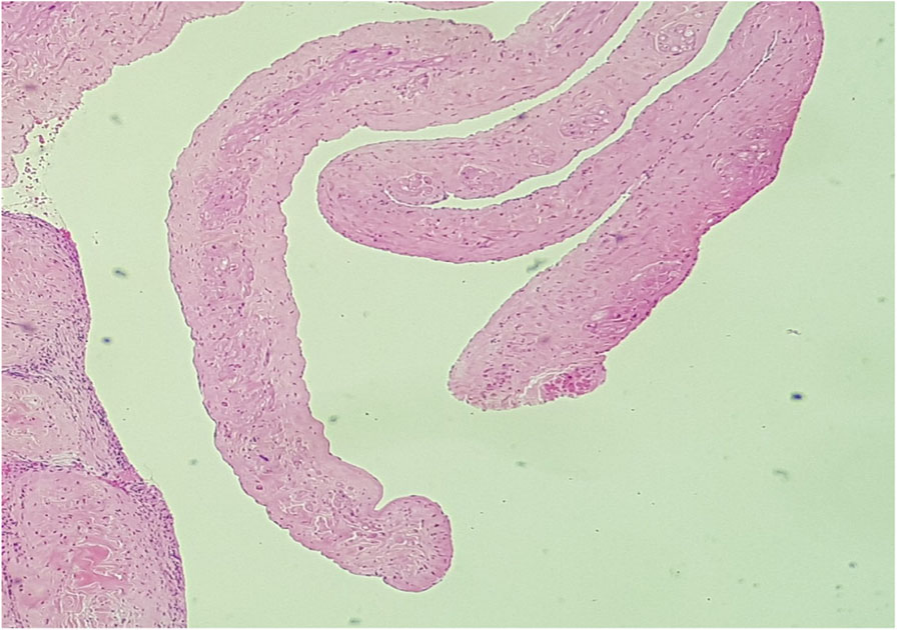
Higher magnification shows these papillary fronds, consisting central avascular core, containing variable amount of collagenous and elastic tissues, surrounded by a single layer of benign endothelial cells (arrow), H&E, X200 magnification

A 27 years-old gentleman with type 1 diabetes mellitus and renal failure secondary to diabetic kidney disease requiring regular haemodialysis via indwelling right internal jugular catheter was admitted for community acquired pneumonia. Clinically, he was febrile but remained comfortable without respiratory distress, and his conditions improved with intravenous antibiotics. He has had multiple episodes of central venous catheter insertion via internal jugular veins while scheduling for arteriovenous fistula procedure. On day 4 of admission, however, he developed respiratory distress with type 1 respiratory failure. A computed tomography pulmonary artery scan revealed distal pulmonary embolism in segmental arteries of right lung, and a soft tissue density mass measuring 3.4cmX3.4cmX2cm within the right atrium. An urgent trans-thoracic echocardiography showed a 3.1cmX3.7 cm pedunculated mass in the right atrium, with the stalk attached to interatrial septum (Fig. 1). The hyperechoic mass appeared to be mobile and round, with stipple in texture and a well demarcated border, features typical of papillary fibroelastoma. His conditions improved after commencement of intravenous heparin therapy. Surgical resection of the intracardiac mass was performed, which revealed a soft, gelatinous mass with flower-like appearance measuring 4x6cm, with a stalk attached to the junction of coronary sinus and interatrial wall (Fig. 2). The tumour was resected completely with the interatrial wall preserved, and histopathological examination revealed typical papillary fibroelastoma (Figs. 3 and 4). The patient’s postoperative recovery was uneventful. Follow-up trans-thoracic echocardiography at 3 months and 1 year did not demonstrate tumour recurrence.

## Discussion

Cardiac papillary fibroelastoma usually occurs sporadically, and it commonly affects 4th to 8th decades of life [1]. The location of tumour typically has a high propensity towards the valvular surface, although it can appear anywhere on any endothelial surfaces, predominantly the left ventricle [1]. In this case, however, papillary fibroelastoma was found at the right atrium without any valvular involvement, a relatively uncommon site for the tumour. The pathogenesis of papillary fibroelastoma remains uncertain. Recently, various reports have described papillary fibroelastoma as neoplasm, hamartoma, organizing thrombi, post-traumatic tumour, and even chronic virus endocarditis [2–4] The microthrombus theory is one of the most commonly recognized explanation which describes the ‘Lambl’s excrescences’, that they originated as small thrombi and later coalesce at the site of endothelial damage [2]. Similarly, our patient has had history of multiple central venous catheter insertion. Endothelial erosions produced by continuous micro-movement of the indwelling catheter with the deposition of fibrin on the thrombogenic catheter surface triggers the development of mural thrombi in right atrium, which serve as nidus for progression of microthrombi and subsequently coalesce into fibroelastoma in this young patient.

Papillary fibroelastomas are most often asymptomatic and diagnosed incidentally on echocardiography or other imaging modalities such as computed tomography scan, during open heart surgery, or even at autopsy. These tumours are generally friable and slow growing. Unlike the unusually large size of papillary fibroelastoma found in our patient, these tumours are typically small tumours [1]. Trans-thoracic echocardiography is often used as a first-line non-invasive assessment to differentiate against vegetation, myxoma and thrombi. Although histologically benign, they can result in life-threatening embolic complications from embolization of tumour fragments into cerebral and coronary arteries resulting in transient ischemic attack, ischemic stroke, myocardial ischemia and sudden cardiac death. These symptoms are more commonly associated with papillary fibroelastoma since they are found predominantly in the left side of the heart. In contrary, right-sided papillary fibroelastomas are most often clinically silent until they become large enough to form a surface thrombus that can cause obstruction to intracardiac blood flow; or intermittent dislodgement of papillary frond fragments and their surface thrombi into pulmonary circulation leading to pulmonary embolism, as evident in this case. The friability and extreme mobility of their tissues often result in high embolic potential.

Therapeutic decisions for papillary fibroelastoma not only depend on its clinical presentations, but also the presence of potential life-threatening embolic events. Generally myxoma necessitates a clear surgical resection margin due to its high rate of recurrence. In contrary, papillary fibroelastoma rarely recurs after resection [5]. In symptomatic patients, particularly with embolic episodes, curative and complete surgical resection of the tumour is strongly warranted in the absence of major contraindication. Symptomatic patients who are surgically unfit for procedure may benefit from long-term anticoagulation therapy to prevent formation of surface thrombi. However, the management for asymptomatic patients with incidentally detected papillary fibroelastoma remains a treatment dilemma. Surgery may be deferred for asymptomatic patients with small tumour, although close monitoring with echocardiography is warranted. We recommend surgical resection of papillary fibroelastoma in asymptomatic patients regardless its anatomical location if the tumour is large(> 1 cm), pedunculated and highly mobile to reduce the risk of potential embolization [5]. The initiation of prophylactic anticoagulation therapy may be beneficial to prevent formation of thrombi and reduce the risk of embolic complications until surgical resection is accomplished.

## Conclusion

We report a rare case of large papillary fibroelastoma located in an unusual location of right atrium which has led to pulmonary embolism due to fragmentation of tumour or surface thrombi owning to its size, friability and mobility. Therapeutic decisions should take into consideration both the presenting symptoms and potential life-threatening embolic complications. Surgical resection of papillary fibroelastoma is curative and carries excellent postoperative outcomes with low recurrence rate. It is hoped that this case report will increase awareness of right atrium intracardiac tumour, particularly papillary fibroelastoma, as a cause of pulmonary embolism especially in patients with indwelling central venous catheter.

## Data Availability

Not applicable.
